# Molecular docking studies of dithionitrobenzoic acid and its related compounds to protein disulfide isomerase: computational screening of inhibitors to HIV-1 entry

**DOI:** 10.1186/1471-2105-9-S12-S14

**Published:** 2008-12-12

**Authors:** Uthaman Gowthaman, Mannu Jayakanthan, Durai Sundar

**Affiliations:** 1 Bioinformatics Centres of BTISnet at Pondicherry University and IIT Delhi, India; 2 Department of Biochemical Engineering and Biotechnology, Indian Institute of Technology (IIT) Delhi, Hauz Khas, New Delhi 110016, India

## Abstract

**Background:**

Entry of HIV-1 into human lymphoid requires activities of viral envelope glycoproteins gp120 and gp41, and two host-cell proteins, the primary receptor CD4 and a chemokine co-receptor. In addition, a third cell-surface protein called protein disulfide isomerase (PDI) is found to play a major role in HIV-1 entry. PDI is capable of mediating thio-disulfide interchange reactions and could enable the reduction of gp120 disulfide bonds, which triggers the major conformational changes in gp120 and gp41 required for virus entry. In this scenario, inhibition of HIV-1 entry can be brought about by introducing agents that can block thiol-disulfide interchange reaction of cell surface PDI. There have been studies with agents that inhibit PDI activity, but the exact mode of binding remains to be elucidated; this might provide insights to develop new drugs to target PDI. This study attempts to perceive the mode of binding of dithionitrobenzoic acid (DTNB), and its structurally related compounds on PDI enzyme.

**Results:**

We performed molecular docking simulation with six different inhibitors (ligand), which includes DTNB, NSC695265, thionitrobenzoic acid, 2-nitro-5-thiocyanobenzoic acid, 2-nitro-5-sulfo-sulfonyl-benzoic acid and NSC517871 into the redox-active site [C37-G38-H39-C40] of the PDI enzyme and the activity was inferred by redox inhibitory models. All ligands showed favorable interactions and most of them seemed to bind to hydrophobic amino acids Ala34, Trp36, Cys37, Cys40, His39, Thr68 and Phe80. The redox inhibitory conformations were energetically and statistically favored and supported the evidence from wet laboratory experiments reported in the literature.

**Conclusion:**

We demonstrated that *in silico *docking experiment can be effectively carried out to recognize the redox inhibitory models of PDI with inhibitor molecules. Interestingly we found that number of docked clusters with each ligand varies in the range of five to eight and conveys that the binding specificity of each inhibitor varies for PDI. We also identified that Cys37 of the enzyme plays an important role in hydrogen bonding with inhibitors. This residue can be considered to being an active site for anti-HIV drug design. Therefore, by inhibiting PDI, one can, not only prevent the viral entry but also circumvent the problem of viral resistance

## Background

The entry of virus into target cell represents one of the most attractive targets in the search for new drugs to treat HIV infection. The entry of HIV-1 into target cells requires the cooperation of the viral envelope glycoproteins gp120 and gp41, and of two host-cell proteins, the primary receptor CD4 and a chemokine co-receptor [1]. Several agents have been developed to target these key regulatory proteins that are essential for HIV replication. Several of them are in clinical trials and one of them has been approved by the FDA for clinical use. Therefore, drugs targeting HIV-1 entry are an exiting prospect in terms of prevention of AIDS. Recently another cell-surface protein was found to be involved in HIV-1 entry, the oxidoreductase protein disulfide isomerase (PDI, E.C. 5.3.4.1) which catalyzes thiol-disulfide interchange reactions [2,3]. It is present mostly in the endoplasmic reticulum and act as oxidase to forms disulfide bonds in nascent proteins and assists in protein folding [4]. It also occurs at the surface of mammalian cells, where it acts as a reductase to cleave disulfide bonds of proteins attached to the cell [5]. Its redox function is based on the presence of two cysteine residues in its active sites Cys-Gly-His-Cys (CXXC). When the cysteine of CXXC bears two cysteinyl thiols, it breaks neighboring disulfide bonds. In the event of HIV-1 entry, the viral glycoprotein gp120 attaches the virus to the cell by binding to its receptor CD4 which also contains disulfide bonds. After CD4 binding, various gp120 domains interact with the enzyme PDI and the chemokine co-receptors forms a PDI-CD4-gp120-chemokine complex. PDI can reach the complex and reduce disulfide bonds in gp120, which causes key conformational changes in gp120 and activate gp41 for the fusogenic potential of the viral envelope [3]. It has been shown that inhibition of HIV-1 entry can be brought about by introducing membrane impermeant sulfhydryl agents that can block the redox function of PDI [2]. These agents will stop the generation of two free thiols in a Gp120 and an oxidized form of CXXC motif in PDI.

It was reported that the membrane-impermeant thiol reagent dithionitrobenzoic acid (DTNB) causes 100% inhibition of soluble PDI activity at 1.0 mM concentration [2,3]. The exact mode of binding interaction is yet to be elucidated and this would give more insights into development of new effective drugs that target PDI activity. Therefore, this necessitates a rational study on the mode of binding of the inhibitors to PDI. This can be achieved by molecular docking studies to determine whether two molecules interact and to find the orientation that maximizes this interaction as well as minimizing the total energy of the interaction complex. Predicting the mode of protein interaction with other molecules promises deduction of protein function and the enhancement of drug discovery. A tangible example can be seen with HIV-1 Protease [6]. The current study attempts to find the mode of binding of DTNB and its related compounds on PDI. The Accelrys Discovery Studio and AutoDock 4.0 [7] were used to study the interaction. Therefore, by inhibiting PDI, one can not only prevent the viral entry, but also circumvent the problem of viral resistance because PDI is a host protein [8].

## Methods

**Figure 1:**
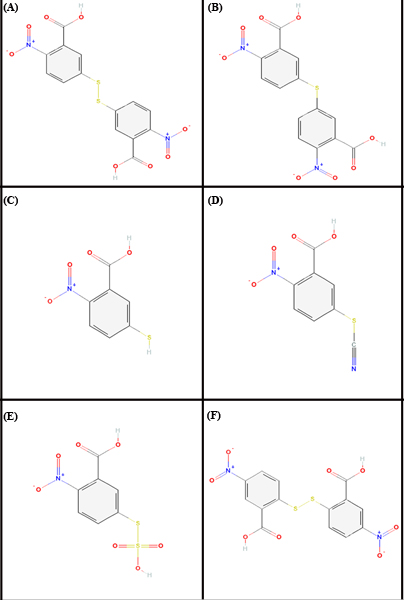
**Two dimensional molecular structures of the ligands/inhibitors**. (A) dithionitrobenzoic acid (DTNB), (B) NSC695265 (5-(3-carboxy-4-nitro-phenyl) sulfonyl-2-nitrobenzoic acid), (C) Thionitrobenzoic acid, (D) 2-nitro-5-thiocyanobenzoic acid, (E) 2-nitro-5-sulfo-sulfonyl-benzoic acid and (F) NSC517871

The set of ligand molecules studied in this work include dithionitrobenzoic acid (DTNB) [PubChem:6254] and its structurally similar bioactive compounds such as NSC695265 (5-(3-carboxy-4-nitro-phenyl) sulfonyl-2-nitrobenzoic acid) [PubChem:393411], Thionitrobenzoic acid [PubChem:123648], 2-nitro-5-thiocyanobenzoic acid [PubChem:92266], 2-nitro-5-sulfo-sulfonyl-benzoic acid [PubChem:128933] and NSC517871 (2-(2-carboxy-4-nitro-phenyl) disulfonyl-5-nitrobenzoic acid) [PubChem:351009]. These ligand molecules were retrieved from NCBI-PubChem Compound database [9,10]. The structure of these compounds is shown in Figure 1. The three dimensional structure of second thioredoxin-like domain of human Protein disulfide-isomerase [PDB: 1X5C] was obtained from Protein Data Bank (PDB) [11,12]. This structure was determined using NMR experimental method [13,14]. The energy of the ligand molecules and target PDI were minimized in Steepest Descent followed by Conjugate Gradient method using Accelrys Discovery Studio (Version 1.7, Accelrys Software Inc.), the most comprehensive suite of modeling and simulation solutions for drug discovery available. Each of the minimization methods were carried out with CHARMm force field.

In order to carry out the docking simulation, we used the AutoDock 4.0 suite as molecular-docking tool [15]. It is suitable software for performing automated docking of ligands to their macromolecular receptors. Typically, the ligands are substrates or drug candidates and the macromolecule is a protein of known three dimensional structures. In this docking simulation, we used semi-flexible docking protocols. In which the target protein PDI was kept as rigid. The ligands being docked were kept flexible, in order to explore an arbitrary number of torsional degrees of freedom in addition to the six spatial degrees of freedom spanned by the translational and rotational parameters. The Graphical User Interface program "AutoDock Tools" was used to prepare, run, and analyze the docking simulations. Kollman united atom charges, solvation parameters and polar hydrogens were added into the receptor PDB file for the preparation of protein in docking simulation. This PDI enzyme structure does not have any water molecules and/or ligands to remove from its PDB file and make a free receptor. Since ligands are not peptides, Gasteiger charge was assigned and then non-polar hydrogens were merged. The rigid roots of each ligand were defined automatically instead of picking manually. The amide bonds were made non-rotatable. All rotable dihedrals in the ligands were assigned with the help of auxiliary program Auto-Tors and were allowed to rotate freely. AutoDock requires pre-calculated grid maps, one for each atom type, present in the ligand being docked and its stores the potential energy arising from the interaction with macromolecule. This grid must surround the region of interest in the macromolecule. In the present study, the binding site was selected based on the amino acid residues, which are involved in redox function and is required for HIV entry into the host cell. Therefore, the grid was centered in the catalytic active region of redox-active Cys-Gly-His-Cys motif and includes all amino acid residues that surround active site. The grid box size was set at 40, 40, and 40 A° (x, y, and z), though it was changed depending on the ligand size. AutoGrid 4.0 Program, supplied with AutoDock 4.0 was used to produce grid maps. The spacing between grid points was 0.375 angstroms.

The Lamarckian Genetic Algorithm (LGA) was chosen to search for the best conformers. During the docking process, a maximum of 10 conformers was considered for each compound. The population size was set to 150 and the individuals were initialized randomly. Maximum number of energy evaluation was set to 250000, maximum number of generations 1000, maximum number of top individual that automatically survived set to 1, mutation rate of 0.02, crossover rate of 0.8, Step sizes were 0.2 Å for translations, 5.0° for quaternions and 5.0° for torsions. Cluster tolerance 0.5A°, external grid energy 1000.0, max initial energy 0.0, max number of retries 10000 and 10 LGA runs were performed. All the AutoDock docking runs were performed in Intel Pentium PD-925 CPU @ 3.0 GHz of HCL infosystem origin, with 2 GB DDR RAM. AutoDock 4.0 was compiled and run under Windows XP operating system.

## Results and discussion

Existing NMR structure of protein disulfide isomerase was downloaded from PDB [PDB: 1X5C]. An examination of this NMR structure for the domain of PDI showed the cysteines in their CXXC motifs (C37-G38-H39-C40) are in the form of free thiols. At the cell surface, this isomerase predominantly acts as reductase leads to the cleavage of disulfide bonds and production of free thiols in the interacting protein (gp120). The criteria which we have followed in the selection of ligands includes only targeting cell surface PDI by using membrane impermeant sulfhydryl inhibitors. Investigational membrane impermeant PDI inhibitors include thiol blockers such as DTNB, NSC695265, thionitrobenzoic acid, 2-nitro-5-thiocyanobenzoic acid, 2-nitro-5-sulfo-sulfonyl-benzoic acid and NSC517871 were retrieved from PubChem database. All the ligands (inhibitors) and PDI structures were confirmed to have minimized energy based on the final potential energy obtained from the results as shown in the Table 1. The potential energy value of all ligands was below -100 kcal/mol. These ligand molecules have the potential to change the original states of it is into a system of binding state in a target PDI molecule when the energy is released. The potential energy of PDI was -7435. 981 kcal/mol. Molecular docking simulations were conducted with the AutoDock 4.0 software suite. 10 Docking runs were performed for each of the six different ligands into redox-active site [C37-G38-H39-C40] of PDI protein. For each docking simulation, the activity inferred by redox inhibitory docked models, in which the docked conformers of ligands and PDI would permit redox active interaction (CXXC motif interaction) and matched with the wet lab experimental observation results of inhibition [2]. To verify the accuracy of the AutoDock 4.0 results, we also considered some top clusters of conformations/orientations in addition to the best scored one. Here, the docking accuracy was evaluated in terms of the root mean square deviation (RMSD) between the docked position and the experimentally determined position for the ligand. In this molecular docking study, prediction was considered successful if the RMSD value is less then 2.0 Å for the best-scored conformation [16]. The redox inhibitory conformations were energetically and statistically validated.

**Table 1:** Minimized energy values for selected ligand molecules

**Molecules**	**Energy levels after Steepest Descent (kcal/mol)**				**Energy levels after Conjugate Gradient (kcal/mol)**			
	
	**Int PE**	**PE**	**VdwE**	**EE**	**Int PE**	**PE**	**VdwE**	**EE**
Dithionitrobenzoic Acid	331.53835	-34.79587	6.27249	-69.98690	-34.79579	-45.38513	6.89156	-77.57011
NSC695265	180.65760	-28.82409	5.10126	-71.60158	-28.82403	-39.42310	6.24088	-81.42580
Thionitrobenzoic acid	79.01217	-17.88183	4.14326	-33.77424	-17.88183	-17.94201	4.03114	-34.15011
2-nitro-5-thiocyanobenzoic acid	46.04640	-21.75786	4.86418	-39.49934	-21.75786	-24.37826	3.72729	-40.29113
2-nitro-5-sulfo-sulfonyl-benzoic acid	71.29484	-14.62018	3.70143	-30.79463	-14.62018	-22.80483	2.65945	-38.26210
NSC517871	200.70255	-62.11036	5.20568	-87.42863	-62.11034	-96.68291	8.13743	-118.83934

### Docking of DTNB into PDI

**Figure 2:**
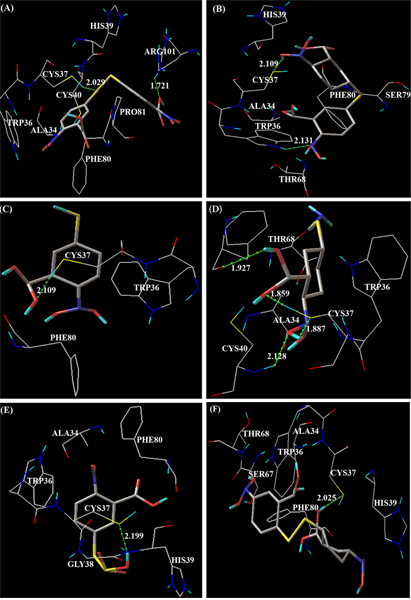
**Representative docking redox-inhibitory models of**. (A) DTNB, (B) NSC695265 (5-(3-carboxy-4-nitro-phenyl) sulfonyl-2-nitrobenzoic acid), (C) Thionitrobenzoic acid, (D) 2-nitro-5-thiocyanobenzoic acid, (E) 2-nitro-5-sulfo-sulfonyl-benzoic acid and (F) NSC517871 (2-(2-carboxy-4-nitro-phenyl) disulfonyl-5-nitrobenzoic acid) into PDI enzyme. The green dot lines denote the hydrogen bonds. All the amino acid residues which involved in molecular interaction are shown in wire frame drawing and colored by atom types in which hydrogen is colored cyan, carbon gray, oxygen red, nitrogen blue, and sulfur yellow. Ligands are shown in stick drawing.

As shown in Table 2, the docking results are ranked according to the ascent of the binding energies for each of the ligands investigated. Docking simulation of DTNB into the redox active site of the PDI produced eight clusters of conformers using RMSD-tolerance of 2.0 Å out of 10 docking runs (Table 2). The conformation of the #1 ranked cluster was favored in that structure and repeated two times out of 10 runs. Inspection of this conformation showed that it could explain the non-redox inhibitory mode of PDI (structure not shown), since docked region was out of redox active site. The conformation of the #5 ranked cluster of DTNB in the PDI was also highly energetically and statistically favored. In which, it was observed that two redox inhibitory docking modes were found in same cluster rank, which would explain the inhibitor effect of the enzyme. In these two conformations, there were, three, two hydrogen bonds formed, respectively (Table 3). The thiol group of Cys37 and backbone-oxygen atom of Phe80 in PDI enzyme interacts with the two different atom of sulfur (S2 and S1) in DTNB to form multiple hydrogen bonds in both the two conformations with a bond length of approximately 2.0 Å, respectively. In addition, side chain nitrogen atom of Arg101 was also involved in the hydrogen bond interaction with O3 atom of DTNB in the lowest binding energy complex of #5 ranked clusters. The DTNB was also made additional van der Waals forces with the residues Ala34, Trp36, His39, Cys40, and Pro81 in the scaling factor around 1.00 Å to further stables the interaction (Figure 2A). The lowest binding energy of the docked complex was 57.26 kcal/mol. The lowest cluster root mean square deviation (RMSD) between the docked conformation and the conformation in the crystal structure was 1.66 Å. The conformations of other clusters of DTNB in PDI were all non-redox inhibitory model. In these instances, DTNB was bound in similar fashion to that seen with cluster rank #1.

**Table 2:** Summary of docking for all six ligands into protein disulfide isomerase

		**Docking statistics (RMSD-tolerance of 2.0 Å)**			
			
**Molecules**	**Number of clusters**	**Cluster rank**	**Lowest binding energy (kcal/mol**	**Number of runs**	**Inferred reactivity**
DTNB	8	1	-2.56	2	Non-redox Inhibitory
		2	+17.08	1	Non-redox Inhibitory
		3	+29.52	1	Non-redox Inhibitory
		4	+37.83	1	Non-redox Inhibitory
		5	+57.26	2	Redox Inhibitory
			+64.98 (2^nd ^sub-rank)		Redox Inhibitory
		6	+76.30	1	Non-redox Inhibitory
		7	+82.38	1	Non-redox Inhibitory
		8	+90.87	1	Non-redox Inhibitory
NSC695265	5	1	-2.81	1	Non-redox Inhibitory
		2	+3.24	1	Non-redox Inhibitory
		3	+5.64	5	Non-redox Inhibitory
		4	+8.43	2	Non-redox Inhibitory
			+9.77(2^nd ^sub-rank)		Redox Inhibitory
		5	+9.61	1	Non-redox Inhibitory
Thionitrobenzoic acid	6	1	+7.33	1	Non-redox Inhibitory
		2	+9.46	4	Redox Inhibitory
			+9.68 (2^nd ^sub-rank)		Redox Inhibitory
		3	+12.48	1	Non-redox Inhibitory
		4	+21.07	2	Non-redox Inhibitory
		5	+34.05	1	Non-redox Inhibitory
		6	+36.25	1	Non-redox Inhibitory
2-nitro-5-thiocyanobenzoic acid	6	1	+10.52	2	Non-redox Inhibitory
		2	+14.16	1	Non-redox Inhibitory
		3	+24.91	2	Non-redox Inhibitory
		4	+25.42	3	Non-redox Inhibitory
		5	+31.31	1	Non-redox Inhibitory
		6	+72.55	1	Redox Inhibitory
2-nitro-5-sulfo-sulfonyl-benzoic acid	8	1	+1.45	1	Non-redox Inhibitory
		2	+3.40	1	Non-redox Inhibitory
		3	+5.56	1	Non-redox Inhibitory
		4	+5.82	1	Non-redox Inhibitory
		5	+6.19	1	Redox Inhibitory
		6	+8.48	3	Non-redox Inhibitory
		7	+23.72	1	Non-redox Inhibitory
		8	+44.52	1	Redox Inhibitory
NSC517871	7	1	-1.12	2	Non-redox Inhibitory
		2	+0.26	1	Non-redox Inhibitory
		3	+2.77	2	Non-redox Inhibitory
			+13.37 (2^nd ^sub-rank)		Redox Inhibitory
		4	+10.25	1	Non-redox Inhibitory
		5	+10.67	1	Non-redox Inhibitory
		6	+11.01	1	Non-redox Inhibitory
		7	+12.32	2	Non-redox Inhibitory

**Table 3:** Molecular interactions of all six ligands into protein disulfide isomerase

**Molecules**	**Hydrogen bond donor**	**Hydrogen bond acceptor**	**Length of hydrogen bond (Å)**	**Residues involved in van der Waals interaction (Scaling Factor = 1.00 Å)**	**Binding free energy (kcal/mol)**
Dithionitrobenzoic acid	LIGAND1::MOL1:S1	PDI: A: PHE80: O		ALA34, TRP36, HIS39, CYS40, PRO81	57.26
	PDI:A:CYS37:HG	LIGAND1::MOL1:S2	2.029		
	PDI:A:ARG101:HH11	LIGAND1::MOL1:O3	1.721		
NSC695265	LIGAND2::MOL1:H11	PDI:A:CYS37:SG	2.109	ALA34, HIS39, THR68, SER79, PHE80	9.77
	PDI:A:TRP36:HE1:	LIGAND2::MOL1:O6	2.131		
Thionitrobenzoic acid 2-nitro-5-thiocyanobenzoic acid	PDI:A:CYS37:SG	LIGAND3::MOL1:O1	2.109	TRP36, PHE80	9.46
	PDI:A:CYS37:HG	LIGAND4::MOL1:O1	1.859	ALA34, TRP36, THR68	72.55
	LIGAND4::MOL1:H7	PDI:A:CYS37:SG	1.887		
	PDI:A:CYS40:HN	LIGAND4::MOL1:O4	2.128		
	LIGAND4::MOL1:H5	PDI: A: PHE80: O	1.927		
2-nitro-5-sulfo-sulfonyl-benzoic acid	LIGAND5::MOL1:H22	PDI:A:CYS37:SG	2.199	ALA34, TRP36, GLY38, HIS39, PHE80	6.19
NSC517871	LIGAND6::MOL1:H9	PDI:A:CYS37:SG	2.025	ALA34, TRP36, HIS39, SER67, THR68, PHE80	13.37

### Docking of NSC695265 into PDI

Docking simulation of NSC695265 (5-(3-carboxy-4-nitro-phenyl) sulfonyl-2-nitrobenzoic acid) into PDI resulted in five clusters of conformers (Table 2). The docked models of all the clusters appeared non-redox inhibitory mode, since docked region of the compound in out side of the redox active site. In detailed inspection of sub ranked cluster conformations, it was observed that the second sub-rank of #4 cluster represented redox inhibitory mode. In this docked complex, side chain NH group of Trp36 and OH11 group of NSC695265 acts as hydrogen bond donors to make multiple hydrogen bonds with sulfur atom of Cys37 and O6 atom of NSC695265, respectively. The bond distance between donors and acceptors atoms of hydrogen bond was in approximately 2.1 Å (Table 3). In addition to hydrogen bonds, residues Ala 34, His39, Thr68, Ser79, and Phe80 were involved in van der Waals interactions further stabilized the ligand (Figure 2B). Among these residues Ala34 and His39 were in similar binding mode as compared to #5 ranked cluster of DTNB docking. The conformations of the #3 ranked cluster of NSC695265 in PDI were generated five more sub-ranked complexes with non-redox inhibitory modes. These conformations had significantly high frequency of non-redox docking occurrences than those of other ranked clusters.

### Docking of thionitrobenzoic acid into PDI

The ten runs of docking simulations of thionitrobenzoic acid to PDI resulted six clusters of conformers. The conformations of the ranked and sub-ranked #2 cluster were highly favored statistically and energetically and repeated four times (Table 2) with the high frequency of occurrences. Analysis of the positions and orientations of the ligand in the docking models in relationship to the location of the redox-avtice site indicating that the first and second sub-ranked conformation of the #2 cluster represented redox inhibitory mode. In these two conformations, there were same atoms involved in hydrogen bond formation. In which, thiol group of Cys37 participated in hydrogen bond formation with the O1 atom of the ligand (Table 3). As a result, conformations were having similar binding energies with similar bond length of 2.1 Å. In addition to Cys37 residue, the thionitrobenzate was held in position by van der Waals forces with the residues Trp36 and Phe80 (Figure 2C). These same residues were participated in the redox inhibitory mode of DTNB in PDI enzyme. The cluster conformers of the other ranked clusters were in non-redox inhibitory modes.

### Docking of 2-nitro-5-thiocyanobenzoic acid into PDI

The results for docking 2-nitro-5-thiocyanobenzoic acid into PDI enzyme are given in Table 2. One redox inhibitory mode was found (cluster #6, Figure 2D) with the 2-nitro-5-thiocyanobenzoic acid, which could explain the redox inhibitory activity of the enzyme. This conformation was energetically favored. The amino acid residues of PDI involved in the interaction with 2-nitro-5-thiocyanobenzoic acid, which were mostly similar to those residues involved in the redox-inhibitory mode of DTNB in PDI (Table 3). In the conformation of #6 ranked cluster, there were four hydrogen bonds formed between the 2-nitro-5-thiocyanobenzoic acid with residues Cys37, Cys40, Phe80 of PDI target with binding energy of 72.55 kcal/mol. In is interesting to note that the redox-active sites of Cys37 and Cys40 were both involved in hydrogen bond formation. In addition, S-H and S atoms of Cys37 itself acts as hydrogen bond donor and acceptor to interact with O1 and O-H7 atoms of ligand in the formation of hydrogen bonds, respectively. This cluster docked conformation was also stabled by van der Waals with Ala34, Trp36 and Thr68.

### Docking of 2-nitro-5-sulfosulfanyl-benzoic acid into PDI

The number of docked clusters observed with 2-nitro-5-sulfo-sulfonyl-benzoic acid for PDI was less frequency of occurrences, since produced seven different clusters with single conformer and one cluster (#6 ranked) with three conformers (Table 2), indicating a decreased specificity of this ligand with PDI. There were two clusters (#5 and # 8 ranked) of conformers appeared as redox-active mode out of eight clusters generated. The lowest binding energy of # 5 ranked clusters was observed with 6.19 kcal/mol and it was significantly lower than the other ligands docked. There was a single hydrogen bond formed between electronegative atoms of sulfur in Cys37 with O-H22 group of 2-nitro-5-sulfosulfanyl-benzoic acid (Figure 2E). The residues Ala34, Trp36, Gly38, His39 and Phe80 were also taken part in van der Waals interactions with quite same as redox-inhibitory mode of DTNB in PDI in the scaling factor around 1.00 Å.

### Docking of NSC517871 into PDI

The docked models of clusters of NSC517871 (2-(2-carboxy-4-nitro-phenyl) disulfanyl-5-nitrobenzoic acid) in PDI all appeared non-redox inhibitory mode, since the ligand was placed far away from the redox-active binding site. In the second sub-ranked conformation of the #3 ranked cluster, NSC517871 was bound to PDI in a fashion similar to the redox inhibitory modes of DTNB, 2-nitro-5-sulfosulfanyl-benzoic acid and NSC695265 in PDI such that it was the quite same amino acid residues involved in the molecular interaction (Table 3, Figure 2F). In the hydrogen bond formation, sulfur atom of Cys37 acts as hydrogen bond acceptors to made interaction with O-H9 group of NSC517871 with bond length of 2.0 Å as approximately similar to 2-nitro-5-sulfo-sulfonyl-benzoic acid docking in concern to both numbers of hydrogen bond and bond length. The van der Waals interactions between the PDI and NSC517871 were observed in the residues of Ala34, Trp36, His39, Ser67, Thr68 and Phe80 in a maximum similar to those observed in the case of 2-nitro-5-sulfo-sulfonyl-benzoic acid in PDI.

As shown in Table 3, it was observed that all the six different ligand molecules participated in hydrogen bond formation with sulphur atom of the Cys37 and/or Cys40 in PDI. In addition to this, sulfur atom in dithionitrobenzoic acid itself act as hydrogen bond acceptor. Sulfur atoms are larger and have a more diffuse electron cloud than oxygen and nitrogen. The O-H^...^S^- ^hydrogen bond strength in cysteine thiolate anion was estimated experimentally as 16.4 ± 2.0 kcal/mol [17]. This makes binding energy of the complex in positive score. Lipinski's rule of five was calculated for all the six ligand molecules that satisfy the 'rule-of-5' and it was found that all the ligand molecules satisfied the rule for potent inhibitors (Table 4).

**Table 4:** Lipinski properties of the docked ligands

**Molecules**	**Mw**	**LogP**	**H-donor**	**H-acceptor**
Dithionitrobenzoic acid	396.354	3.5	2	8
NSC695265	364.288	3.2	2	8
Thionitrobenzoic acid	199.185	1.9	1	4
2-nitro-5-thiocyanobenzoic acid	224.193	1.5	1	5
2-nitro-5-sulfo-sulfonyl-benzoic acid	279.247	0.3	2	7
NSC517871	396.351	3.5	2	8

## Conclusion

In this work, a molecular docking simulation study was undertaken to investigate the binding mechanism of DTNB and its structurally similar membrane impermeable inhibitors such as DTNB, NSC695265, thionitrobenzoic acid, 2-nitro-5-thiocyanobenzoic acid, 2-nitro-5-sulfo-sulfonyl-benzoic acid and NSC517871 to the protein disulfide isomerase enzyme to enable the finding of potential anti-HIV drugs (namely, the development of inhibitors of surface associated PDI). We determined the energetically favored docking sites for the inhibitors. Our docking results explain that the numbers of clusters with each ligand vary in the range from five to eight for PDI enzyme (Table 2), indicating that the binding specificity of each ligand is varying in PDI. In this study, the hydrogen bond makes important contributions to the interactions between ligand and enzyme. From the frequency of residue's occurrence in the formation of hydrogen bonding, it is evident that Cys37 plays an important role due to the presence of S-H group and it acts as both hydrogen bond donor and acceptor. And Trp36, Cys40, Phe80, Ag101 also take part in the hydrogen bonding with comparatively high frequency. This information could be supportive for new drug design for AIDS, in which these potential inhibitors should interact strongly with above-mentioned residues. Cys37 is of great concern in anti-HIV drug design, which is believed to be one of redox-active site in PDI [2]. It is significant to have this Cys37 S-H group locked such that its reduction function is depressed. Therefore the above-mentioned inhibitors will block the cleavage of disulfide bond in gp120, leading to inactive gp41 and resistance to the entry of HIV. It was observed that there were maximum of four hydrogen bonds formed between PDI and the inhibitors used (Table 3). Besides hydrogen bonding, van der Waals interactions were also taking part in the stabilizations of inhibitors binding with high frequency of residues such as Ala34, Trp36, Cys40, His39, Thr68 and Phe80 in PDI. The redox-inhibitory mode of all six inhibitors with PDI was consistent with the laboratory experimental result of Ryser et al [2].

## Competing interests

The authors declare that they have no competing interests.

## Authors' contributions

UG, MJ and DS designed the methods and experimental setup. UG and MJ carried out the implementation of the various methods. MJ and DS wrote the manuscript. All authors have read and approved the final manuscript.
